# Editorial: Brain health: risk, resilience and reserve

**DOI:** 10.3389/fpsyg.2024.1386516

**Published:** 2024-03-25

**Authors:** Michelle E. Kelly, Joanna McHugh Power, Mario A. Parra, Caoimhe Hannigan

**Affiliations:** ^1^Department of Psychology, National College of Ireland, Dublin, Ireland; ^2^Department of Psychology, Maynooth University, Maynooth, County Kildare, Ireland; ^3^Psychological Sciences and Health, University of Strathclyde, Glasgow, United Kingdom

**Keywords:** cognitive health, aging, resilience, neuroplasicity, brain health, cognitive reserve

Research on brain health is imperative as we consider increasing life expectancy and the increasing prevalence of cognitive decline with advancing age. Risk and protective lifestyle factors driving brain health and reduced risk of cognitive decline have been identified (Livingston et al., 2020), but we have yet to fully understand all factors and contexts that contribute to observed variability in brain health outcomes (Boyle et al., 2018; Stern et al., 2020). This Research Topic, Brain Health: Risk, Resilience, and Reserve, provides insight into current trends and perspectives on the promotion of brain health across the lifespan, with a helpful focus on how research might be translated into recommendations for practice. The articles included in this Research Topic advance our understanding on topics such as cognitive reserve, brain reserve, resilience, and brain health.

When considering risk and protective lifestyle factors for brain health, an important question is whether people are indeed aware of behaviors that impact their brain health. The research by Dukelow, Lawrence et al. addresses this question with reference to data from the Five Lives Brain Health Ireland Survey (FLBHIS) in Ireland. The study shows that there is a general lack of awareness across the population about preventative lifestyle factors impacting dementia risk. In this study, knowledge of—and exposure to—risk factors varied depending on age, gender, and education levels. The paper by Dukelow, Vassilev et al. also examined data from the FLBHIS, and identified multiple barriers that reduce the likelihood of people adapting their lifestyle to positively impact brain health. Lack of motivation tended to influence more practical factors like physical activity, while more nuanced emotional factors tended to be barriers to social engagement.

While social engagement (Penninkilampi et al., 2018) and loneliness (Harrington et al., 2023) are both related to brain health, loneliness is not entirely contingent on social relationships (Masi et al., 2011; Kelly et al., 2017). Franco-O'Byrne et al. delve further into the topic of loneliness and cognitive function by providing an overview of how social adaptation may buffer against the negative effects of loneliness on brain health. This research, based on data from participants in Chile, offers interesting ideas on the potential for brain health preservation via adaptive social behaviors.

For older adults living in more isolated geographical areas, being able to drive can provide greater opportunities for social contact, and driving cessation is a risk factor for social isolation (Qin et al., 2020), which in turn predicts dementia (Kuiper et al., 2015). Murphy et al. recruited a sample of cognitively healthy older adults from the U.S. to explore whether cognitive and brain reserve predicted changes in driving behavior. They found that those with lower levels of brain and cognitive reserve restricted or adapted their driving behavior more than those with higher reserve. Restricted driving behavior might have a further knock-on effect on cognitive reserve if this results in lower levels of social and cognitive stimulation.

If adaptations in driving behavior are necessary for safety reasons, compensatory avenues for social or cognitive stimulation could be explored. Interventions that involve structured cognitively stimulating activities, for instance, can be beneficial in improving cognitive outcomes and psychological wellbeing for those experiencing cognitive decline (Verghese et al., 2003; Najar et al., 2019). Dinius et al. describe their recent research on Reminiscence Therapy (RT) interventions in Ireland and discuss how RT can be combined with other activities (like walking) to support brain health and wellbeing in those with and without dementia. Cognitive interventions are also the focus of the systematic review by Fava-Felix et al., but they focused on computerized cognitive training post-stroke. The review considers the impact of variables such as education level on intervention outcomes, and contributes to the discussion around the importance of early intervention.

This Research Topic brings together research from around the world to provide important insights into factors that promote brain health in a variety of contexts and for a diverse range of individuals. Brain health awareness and education, potential interventions, and observational studies on correlates of brain health are all presented. The included studies and reviews offer practical recommendations for researchers, practitioners, and anyone interested in keeping their brain healthy. Most crucially, the research demonstrates paths forward for further research in how to best promote, maintain, and intervene on brain health, both in general populations and among those with cognitive impairment and dementia.

## Author contributions

MK: Conceptualization, Resources, Writing – original draft, Writing – review & editing. JM: Writing – original draft, Writing – review & editing. MP: Writing – original draft, Writing – review & editing. CH: Conceptualization, Resources, Writing – original draft, Writing – review & editing.
